# Description of CD8^+^ Regulatory T Lymphocytes and Their Specific Intervention in Graft-versus-Host and Infectious Diseases, Autoimmunity, and Cancer

**DOI:** 10.1155/2018/3758713

**Published:** 2018-08-05

**Authors:** Martha R. Vieyra-Lobato, Jorge Vela-Ojeda, Laura Montiel-Cervantes, Rubén López-Santiago, Martha C. Moreno-Lafont

**Affiliations:** ^1^Departamento de Inmunología y Departamento de Morfología, Escuela Nacional de Ciencias Biológicas, Instituto Politécnico Nacional, Prolongación de Carpio y Plan de Ayala S/N, Colonia Santo Tomás, Miguel Hidalgo, 11340 CDMX, Mexico; ^2^Unidad Médica de Alta Especialidad, Centro Médico Nacional La Raza, Instituto Mexicano del Seguro Social, Seris y Zaachila S/N, Colonia La Raza, Atzcapotzalco 02990 CDMX, Mexico

## Abstract

Gershon and Kondo described CD8^+^ Treg lymphocytes as the first ones with regulating activity due to their tolerance ability to foreign antigens and their capacity to inhibit the proliferation of other lymphocytes. Regardless, CD8^+^ Treg lymphocytes have not been fully described—unlike CD4^+^ Treg lymphocytes—because of their low numbers in blood and the lack of specific and accurate population markers. Still, these lymphocytes have been studied for the past 30 years, even after finding difficulties during investigations. As a result, studies have identified markers that define their subpopulations. This review is focused on the expression of cell membrane markers as CD25, CD122, CD103, CTLA-4, CD39, CD73, LAG-3, and FasL as well as soluble molecules such as FoxP3, IFN-*γ*, IL-10, TGF-*β*, IL-34, and IL-35, in addition to the lack of expression of cell activation markers such as CD28, CD127 CD45RC, and CD49d. This work also underlines the importance of identifying some of these markers in infections with several pathogens, autoimmunity, cancer, and graft-versus-host disease as a strategy in their prevention, monitoring, and cure.

## 1. Introduction

In general, CD8^+^ Treg lymphocytes have been characterized as a heterogeneous population consisting of lymphoid cells that express certain surface markers depending on their inhibition activity and the microenvironment they are found in [1].

In 1970, Gershon and Kondo described CD8^+^ Treg lymphocytes for the first time when they published the results of experiments using mice. The study described a population of lymphocytes from bone marrow responsible for tolerance. These cells were originally called “suppressor T lymphocytes.” In their work, the researchers proved the cross-reactivity of related antigens by immunizing mice, first using sheep erythrocytes and then horse erythrocytes. The treatment induced tolerance to horse red blood cells in mice that had been immunized with high levels of sheep red blood cells. This tolerance was proven to be mediated by thymic cells [2]. They later proved the regulatory role of peripheral thymocytes, specifically those located in the spleen [3]. The study of these cells was further developed in 2007 under the concept of CD8^+^ Treg cells in the context of some viral infections and development of some tumors. These works established the indirect importance of IFN-*γ* in the induction of their regulatory activity through molecules as indoleamine 2,3-dioxygenase (IDO) [4].

It is currently known that CD8^+^ Treg lymphocytes have an inhibitory effect through soluble factors or cell-cell contact. In murine and human models, different works have described a number of regulatory mechanisms mediated by CD8^+^ Treg lymphocytes: (a) direct death of target cell [5, 6], (b) negative signaling through CTLA-4 or PD1 when interacting with the antigen-presenting cell [7], and (c) release of immunosuppressive cytokines as IL-10 and TGF-*β* [8, 9]. The suppressor effect is evident when CD8^+^ Treg lymphocytes are able to inhibit the proliferation of effector CD4^+^ and CD8^+^ effector T lymphocytes [10]. The immunosuppressive effect of CD8^+^ Treg lymphocytes is likely to be beneficial by reducing the severity of the inflammatory response present during the development of the graft-versus-host disease (GVHD) or autoimmune diseases. On the other hand, it would be beneficial to decrease the CD8^+^ Treg population in diseases such as cancer or infections where they participate in the evasion of the immune response. Proving this effect would shed light on its application as preventive or healing cell therapy.

**Figure 1:**
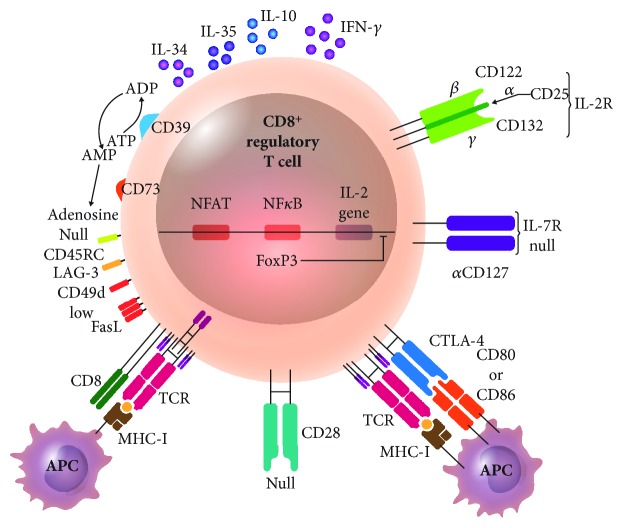
CD8^+^ Treg lymphocyte. CD8^+^ Treg lymphocytes have different suppression mechanisms of cell activation and survival, using their own molecule expression: IL-2 receptor *α*-chain (CD25), IL-2 and IL-15 receptor *β*-chains (CD122), inhibitory receptor CD152 or CTLA-4, ectoenzymes CD39 and CD73 degrading ATP to ADP (CD39) and AMP to adenosine (CD73), an MHC-II-binding molecule called LAG-3 (lymphocyte activation gene-3), and the apoptosis-inducing molecule FasL. This T cell subset expresses low or absent costimulatory receptor CD28 and the IL-7 receptor *α*-chain (CD127), the cellular activation molecule CD45RC, and the integrin CD49d and releases cytokines as IL-10, IL-34, IL35, and IFN-*γ*; transcription factor FoxP3 inhibits IL-2 gene transcription. APC: antigen-presenting cell; CTLA-4: cytotoxic T-lymphocyte antigen 4.

The expression of surface molecules acting as cell markers helps to phenotypically identify CD8^+^ Treg lymphocytes. Phenotypic markers include the high expression of the IL-2 receptor *α*-chain CD25 and expression of CD122 (IL-2 and IL-15 receptor *β*-chains), adhesion molecule CD103, ectoenzymes CD39 and CD73, the inhibition receptor CD152 or CTLA-4 (cytotoxic T lymphocyte-associated molecule-4), an MHC-II-binding molecule called LAG-3, and the apoptosis-inducing molecule FasL. The soluble molecules that CD8^+^ Treg lymphocytes can express are FoxP3, IFN-*γ*, IL-10, IL-34, and IL-35. The absence of activation markers is also studied when looking for CD8^+^ Treg lymphocytes. The costimulation molecule CD28, the IL-7 receptor *α*-chain (known as CD127), the cell activation molecule CD45RC, and the integrin CD49d are absent or show low expression [10] (Figure 1).

### 1.1. Surface Markers of CD8^+^ Treg Lymphocytes

The overexpression of CD25, widely described in CD4^+^ Treg lymphocytes, indicates the presence of a regulatory activity, inhibiting the proliferation of effector lymphocytes in competition for IL-2. Given the high expression of CD25 in the membrane of Treg lymphocytes, the latter obtain most of the cytokine, leaving effector T lymphocytes without the supply of this growth factor. For its part, marker CD25 is commonly sought together with transcription factor FoxP3 [11]. In CD8^+^ Treg lymphocytes, it is unclear whether CD25 subtracts IL-2 from the medium as a regulatory mechanism. However, CD8^+^CD25^+^ Tregs are present in both human and mouse and are very sensitive to IL-2 to proliferate compared to T effectors and capable of inhibiting the proliferation of effector T cells [12].

On the other hand, a subset of CD8^+^CD122^+^ Treg lymphocytes in mice has been observed to be efficient in the suppression of allogeneic, autoimmune, and antitumor responses. Additionally, CD8^+^CD122^+^ T cells express large amounts of IL-15 receptor *α*-chain (IL-15RA). The *β*- (CD122) and *γ*- (CD132) chains are overexpressive and common for CD25 and IL-15R*α*; however, CD25 is absent in those cells. Therefore, the distinctive molecule is CD122 and not CD25. That is why these lymphocytes consume IL-15 to proliferate and not IL-2 [13]. The expression of CD122 is associated with memory lymphocytes [13, 14]. Because nonregulatory memory lymphocytes can also express CD122, the presence of PD-1 is evaluated to confirm that it is CD8^+^CD122^+^ Treg [15]. Apparently, TCR-MHC-I is a mechanism of interaction between these lymphocytes and the target cells [16], and CD8^+^CD122^+^ cells regulate through IL-10 [17].

When CD8^+^CD122^+^ T cells are eliminated from mice, there is a growth of specific tumor T cells and infiltration of effector/memory T cells in the tumor [15, 18]. In mice, marker CD122 is exclusive of CD8^+^ Treg lymphocytes and is absent from CD4^+^CD25^+^ Treg lymphocytes [19]. CD122 works as an IL-15 receptor, which promotes survival and proliferation of CD8^+^ Treg lymphocytes, so that the transfer of CD8^+^CD122^+^ T lymphocytes, along with the administration of recombinant IL-15, promotes its regulatory activity, extending the survival of mice after pancreas transplant [18]. Indeed, in mice, CD122 has made a difference between CD4^+^ and CD8^+^ Treg lymphocytes since, as it has been already stated, the first often express high levels of CD25 while the latter exhibit elevated levels of CD122. For their part, CD8^+^CD122^+^ Treg lymphocytes are related to the success of allogeneic transplant via the induction of apoptosis among alloeffector T lymphocytes and thus inhibiting transplant rejection [20].

In mice, CD8^+^CD122^+^ T cells are comparable with CD8^+^CXCR3^+^ T cells in humans since they release IL-10 and suppress IFN-*γ* production by CD8^+^CXCR3^−^ effector T cells [21].

Also known as LFA-1, CD103 is an adhesion molecule present in T lymphocytes bound to E-cadherin from the parenchymal epithelial tissue or mucous membranes. This molecule promotes retention of Treg lymphocytes in such tissues in areas expressing E-cadherin where the regulation of immune response is needed. This is highly useful to identify CD8^+^ Treg lymphocyte subpopulations according to their location [22]. It must be considered that molecule CD103 does not provide an exclusive regulatory function to CD8^+^ Treg lymphocytes given that CD8^+^ effector T lymphocytes also express it [23, 24].

Ectoenzymes CD39 and CD73 are found on the cell surface of lymphocytes and other cell lines. While CD39 produces ADP and AMP via ATP dephosphorylation, CD73 catabolizes AMP to produce adenosine, which inhibits T lymphocyte response and has an anti-inflammatory effect. The regulatory activity of adenosine starts after it is bound to any of its four receptors: A1, A2A, A2B, and A3. Its effect is greater when bound to receptor A2A. Even though the pathway through which adenosine signals when it is bound to its receptor, *in vitro* studies have found that CD73 inhibits the proliferation of effector T lymphocytes in mice; such effects have been proven in CD4^+^ Treg lymphocytes. Because these markers were later found in human CD8^+^ Treg lymphocytes, they are considered therapeutic targets in therapy against cancer [25–27].

Cytotoxic T lymphocyte antigen-4 (CTLA-4, CD152) blocks the production of IL-2, the expression of IL-2R, and the cell cycle of activated T lymphocytes [28]. CTLA-4 antagonizes CD28 and prevents CD28-CD80/CD86 interaction like an inhibition mechanism [29]. Also, when there is CTLA-4 engagement, the membrane-proximal region of the CTLA-4 cytoplasmic domain delivers a tyrosine-independent signal that inhibits T cell activation, another inhibition mechanism by CTLA-4 [30–32]. Recent works propose a different CTLA-4 suppressor mechanism that involves the capture and depletion of its ligands, CD80 and CD86, from antigen-presenting cells by transendocytosis. During the process, CD80/CD86 are transferred into CTLA-4-expressing cells. Therefore, not only does CTLA-4 uptake its ligands and internalize them but also is likely to degrade them [33–35]. A reduced costimulation in T lymphocytes also reduces positive signals between them and antigen-presenting cells that promote the maturation of the latter. This event occurs in the infiltration of T cells in some types of cancer [28, 36, 37]. The subpopulations of Treg CD8^+^CTLA-4^+^ suppress the immune response against tumor, inhibiting the proliferation of effector T lymphocytes, where they can participate in the regulatory mechanism of IL-35 [38] and are also able to inhibit dependent allogeneic responses [39].

For its part, LAG-3 (lymphocyte activation gene 3) is a molecule with a similar structure to CD4. Because of this similarity, it competitively binds to MHC-II molecules with higher affinity than CD4. When it binds to MHC-II in antigen-presenting cells, it signals in a negative way, unlike CD4 does [40–42]. Therefore, LAG-3 interacts with the TCR-CD3 complex and inhibits its signaling [43]. The interaction between LAG-3 and MHC-II inhibits the activation and proliferation of CD4^+^ and CD8^+^ T cells and the production of cytokines from a Th1 subset [44]. This immune system suppression molecule acts against tumors by blocking them with an antibody, restoring the immune response [45]. Finally, its presence in regulatory cells can decrease the severity of autoimmune diseases [46].

FasL is a molecule involved in the induction of apoptosis of a target cell, a mechanism used by CD8^+^ Treg lymphocytes to kill effector T cells in a direct cytotoxicity. For this regulatory mechanism to work, it is essential that the Treg lymphocyte and the target cell express FasL and Fas, respectively [47, 48].

The characterization of CD8^+^ Treg lymphocytes via the detection of membrane molecules that identify them should be complemented with the research on soluble molecules they express.

### 1.2. Soluble Molecules

FoxP3 is an intracellular DNA-binding protein that prevents transcription and probably involves the direct repression of NF-AT-mediated transcription [4, 49, 50]. It was initially described in scurfy mice that do not express FoxP3. Studies found that CD4^+^ T lymphocytes in scurfy mice were chronically activated, expressing high levels of several activation markers and cytokines ex vivo. This lymphocyte hyperactivation phenotype was refractory to inhibition with a number of drugs, specifically immunosuppressants cyclosporine A and rapamycin [51]. The phenotype of these mutant mice is similar to the one observed in CTLA-4-deficient mice, indicating that FoxP3 is an important regulator of T lymphocyte activation [52]. In CD4^+^ Treg lymphocytes, FoxP3 is a suppression marker of cell activation and thus used as identification marker. For CD8^+^ Treg lymphocytes, the role of FoxP3 is not so clear since it is only expressed in less than 5% of CD8^+^ T lymphocytes [53]. However, populations of CD8^+^ Treg cells expressing FoxP3 are important immune-suppressors during chronic or asymptomatic infections caused by suboptimal amounts of the infectious agent [54]. They also play this role during GVHD and skin transplantation [55].

The proinflammatory cytokine IFN-*γ* polarizes Th1 immune cell response and has been observed to play another role in CD8^+^ Treg lymphocytes. When producing IFN-*γ*, these lymphocytes induce IDO production by dendritic and endothelial cells [4, 56]. This enzyme is responsible for catabolizing tryptophan amino acid. This amino acid is essential to lymphocyte proliferation after activation; therefore, the presence of IDO leads to a decrease in circulating tryptophan levels, restricting the proliferation of activated effector lymphocytes [57]. The single nucleotide polymorphisms (SNPs) of the IDO enzyme are related to autoimmune diseases such as systemic sclerosis [58].

The anti-inflammatory cytokine IL-10 is considered a characteristic molecule of CD4^+^ Treg lymphocytes. Besides mediating the anti-inflammatory regulatory action, it is added to cultures in order to induce CD4^+^ Treg lymphocytes *in vitro*. A similar observation has been made in CD8^+^ Treg lymphocytes, further proving that IL-10 is an evidence of the regulatory function of these cells [59]. For example, IL-10 produced by CD8^+^ Treg lymphocytes inhibits CD4^+^, Th1, and Th2 cell proliferation [60, 61]. In addition, IL-10 suppresses the cytotoxic activity of cytotoxic T lymphocytes by the reduction of MHC-I expression in target cells [62].

A Treg-specific cytokine, IL-34, has an immunosuppressive function and is involved in the maturation of immunoregulatory macrophages during immunological tolerance processes as pregnancy and the inhibition of rejection in solid organ transplantation [63, 64]. The cytokine carries out its regulatory function when it is recognized through the Fms receptor, which it shares with the macrophage colony-stimulating factor (M-CSF) [65]. Additionally, IL-34 has been found to be involved in the regulation of several subpopulations of tissue resident macrophages, including Langerhans cells and microglia [66].

Another cytokine, IL-35, inhibits the maturation of dendritic cells and the proliferation of CD4^+^ and CD8^+^ T cells and the Th1 polarization of CD4^+^ T lymphocytes [67]. Specifically, this cytokine is involved in the suppressive role of CD8^+^ Treg cells in tumors, synergizing with CTLA-4 and avoiding the potentiation of an antitumor immune response [38].

TGF-*β* (transforming growth factor) is an immunoregulatory cytokine that can be expressed in 3 isoforms: TGF-*β*1, TGF-*β*2, and TGF-*β*3, depending on the tissue and the stage of development. It promotes CD8^+^ effector T lymphocyte apoptosis through SMAD-2 signaling and the upregulation of proapoptotic protein Bim [68]. The TGF-*β*-producing CD8^+^ Tregs are able to suppress autoimmune responses very efficiently [69, 70]. It is known that TGF-*β* acts on antigen-presenting cells as dendritic cells decreasing the expression of costimulation and MHC-I molecules and effector T lymphocytes by inhibiting their proliferation. This has been a mechanism described in the evasion of the antitumor immune response [71, 72].

### 1.3. Absence of Activation Molecules

As previously stated, CD8^+^ Treg lymphocytes are characterized by the presence of molecules used as markers to detect and identify these cells. However, it should also be considered that CD8^+^ Treg lymphocytes lack the expression of certain molecules associated to activation and are present in effector T cells. In mice and human, marker CD28 is scarcely expressed in the thymus and has a reduced expression in peripheral blood cells with anti-inflammatory cytokine production; therefore, it is considered that some subpopulations of CD8^+^ Treg could be CD28^low^ [1]. The absence of CD28 in human T lymphocytes correlates with two biological events: cell senescence [73, 74] and extended exposure to antigens [75]. Because of that, there is an increase in CD8^+^CD28^−^ T lymphocyte population during chronic inflammatory processes and in elderly subjects. These cells are produced from CD8^+^ T lymphocytes that have repeated antigen stimulation [76]. This explains the fact that elderly subjects show higher concentrations of these lymphocytes. CD8^+^ T lymphocytes are also unable to proliferate once they are induced to differentiate into CD28^−^ cells [77, 78]. They express regulation molecules that are present in CD4^+^ Treg lymphocytes as CD39, CTLA-4, and CD25. In addition, studies have proven that they are able to inhibit effector CD4^+^ and CD8^+^ effector T lymphocytes. These lymphocytes are considered Treg and able to inhibit a Th1-type response [10, 79–81].

The surface marker CD127 (IL-7 receptor *α*-chain) is also absent from CD8^+^ Treg lymphocytes, recovering its levels of expression in effector and memory cells [82–85] but not in FoxP3^+^ and those that are likely to be regulatory [85]. There is evidence that CD127 is absent from CD8^+^ Treg lymphocytes. This was proven *in vitro* when differentiating naïve CD8^+^ T lymphocyte with TGF-*β* and IL-2 and obtaining lymphocytes with suppressor action expressing CD127^−^CD25^hi^FoxP3^hi^ markers [86]; however, these lymphocytes are not so helpful. In humans and mice, CD4^+^ and CD8^+^ Treg lymphocytes expressing CD25^+^FoxP3^+^ exhibit low concentrations or absence of CD127, unlike effector T cells. This difference is more evident in humans [12].

A T cell activation marker, CD45RC, is absent or found at low concentrations in CD8^+^ Treg lymphocytes involved in solid organ transplant acceptance by IL-34 production [4]. The isoform of CD45, CD45RC, is a transmembrane protein-tyrosine phosphatase that belongs to the Src kinase family. It is essential to signal transduction after T cell receptor activation and is present in rats, mice, and humans [4, 87–92].

Finally, CD49d is a surface molecule expressed at low levels in CD8^+^ Treg lymphocytes. Although the role these lymphocytes play remains unclear, one of their subpopulations can induce apoptosis in activated T lymphocytes through FasL-Fas interactions [48].

### 1.4. Participation of CD8^+^ Treg Lymphocytes in Infection, Autoimmunity, Cancer, and GVHD

Membrane, intracellular, and secretory originating molecules from cells previously mentioned have allowed for the characterization and identification of Treg lymphocytes. Additionally, such molecules confer a suppressant activity upon the activation of other cell populations. In literature, CD8^+^ Treg lymphocytes have been described as key elements in a number of pathologies, including infectious and autoimmune diseases, cancer, and GVHD (Table 1).

### 1.5. CD8^+^ Treg Lymphocytes in Infectious Diseases

**Figure 2:**
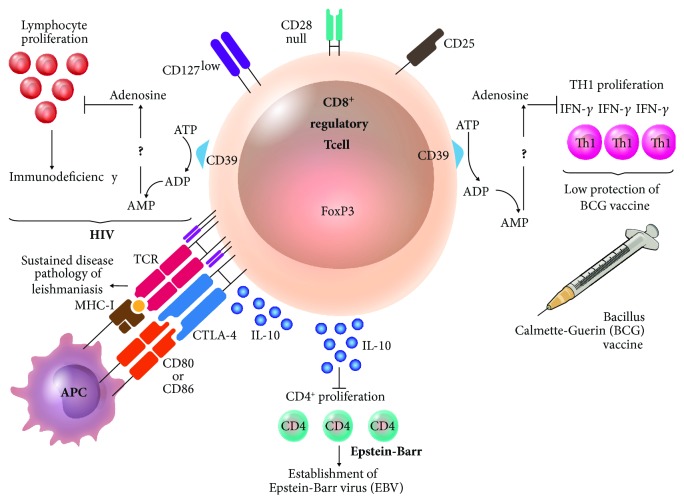
Participation of CD8^+^ Treg lymphocytes in infectious diseases. In an infection with human immunodeficiency virus (HIV), CD8^+^ T lymphocyte has a phenotype CD28^−^CD127^lo^CD39^+^ inhibiting lymphocyte proliferation, which is probably related to the immunodeficiency shown during the disease. In a parasitic infection as leishmaniasis, the persistence of the parasite partly depends on the existence of CD8^+^ Treg lymphocytes expressing CTLA-4 and producing IL-10, which results in the prevalence of the disease. During immunosuppression situations, there is an increase in the population of IL-10-producing CD8^+^FoxP3^+^ Treg lymphocytes that inhibit CD4^+^ T cell proliferation, promoting infection by Epstein-Barr virus. The low protection of bacillus Calmette-Guérin vaccine is attributed factors as CD8^+^CD25^+^CD39^+^ Treg lymphocytes that inhibit the proliferation of CD4^+^ T lymphocytes producing Th1 cytokines as IFN-*γ*, necessary to activate other cell lines against mycobacteria.

In infectious diseases, CD8^+^ Treg lymphocytes reduce immune response against pathogens, which is beneficial to prevent tissue damage caused by an exacerbated response. In contrast, it can also participate in the evasion of host immune response against the pathogen. As an example, the mycobacteria have coexisted with humans for a long time, as *M. tuberculosis*. These bacteria possess different evasion strategies, like the capacity to induce suppressant activity of the immune response mediated by CD8^+^CD25^+^FoxP3^+^CD39^+^ Treg lymphocytes. These lymphocytes, found at higher levels during mycobacteriosis, are able to suppress the proliferation of Th1 (proinflammatory type 1 T helper cells) that produces IFN-*γ*, necessary to activate other cells against mycobacteria. In addition, the measurement of IFN-*γ* has been used in the diagnosis and monitoring of patients. It has recently been observed that vaccination with bacilli Calmette-Guérin induces an increase in CD8^+^ Treg lymphocyte population, which has been related to the low protective action of the vaccine against *M. tuberculosis* [93, 94] (Figure 2).

In individuals coinfected with hepatitis C and human immunodeficiency viruses, the TGF-*β* produced by CD8^+^ Treg lymphocytes reduces the levels of hepatitis C virus-specific effector T lymphocytes. This effect is reversed by blocking TGF-*β* and IL-10 produced by Tregs [95]. Additionally, it has been reported that, during HIV infection, the levels of CD8^+^CD28^−^CD127^lo^CD39^+^ Treg lymphocytes are increased with respect to those found in healthy subjects; CD73 is less abundant [96]. The levels are reduced after administering the antiretroviral treatment to the patients. The Tregs observed in HIV patients are antigen-specific and inhibit the proliferation of peripheral-blood lymphocytes. These observations suggest that the suppressant activity of Treg lymphocytes is one of the factors affecting the immune function in HIV patients [97] (Figure 2).

Although the cytomegalovirus can coexist with the human in a subclinical way, it is of great importance in the production of CD8^+^ T lymphocyte arrays in adult age. This is because studies in adults have found that cytomegalovirus epitope-specific CD8^+^ T lymphocytes constitute a high percentage (33%, approximately) of the total CD8^+^ T lymphocytes, which might compromise the response against other pathogens [75]. A high ratio of these lymphocytes is probably CD28^−^, given that, as it was mentioned before, the absence of CD28 indicates senescence and repeated stimulation with persistent antigens.

Also, CD8^+^ Treg lymphocytes are key to the infection process in transplant patients who are under immunosuppressant conditions due to conditioning chemotherapy previous to transplant and subsequent treatment with immunosuppressants to prevent transplant rejection and GVHD. The levels of IL-10-producing CD8^+^ Treg lymphocytes in transplant patients are higher than those in healthy subjects, which agrees and seems to be associated to the presence of opportunistic pathogens as the Epstein-Barr virus, caused by the inhibition of effector CD4^+^ T lymphocyte proliferation [98] (Figure 2).

In parasitic infections, CD8^+^ regulatory T lymphocytes have been found in visceral leishmaniasis patients who express CTLA-4 and produce IL-10 [99] (Figure 2). When dermal sequelae are caused by *Leishmania donovani* infection, the percentage of CD8^+^CD28^−^T lymphocytes is increased and only restored after treatment [100].

### 1.6. CD8^+^ Treg Lymphocytes in Autoimmune Diseases

**Figure 3:**
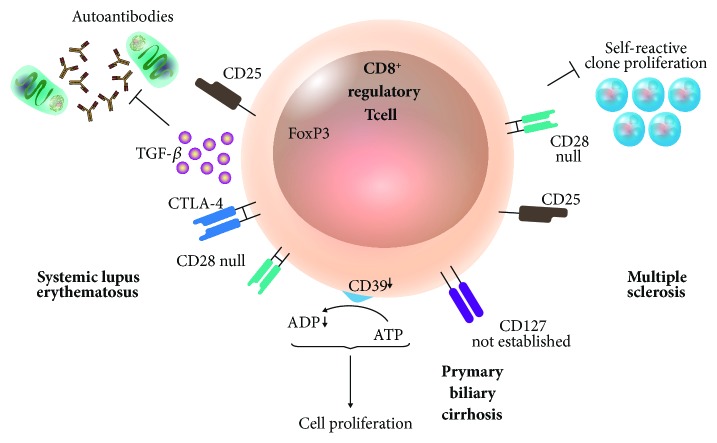
Participation of CD8^+^ Treg lymphocytes in autoimmune diseases. During SLE, antibody production is eliminated thanks to the suppressive activity of TGF-*β*-producing CD8^+^CD25^+^FoxP3^+^ Treg lymphocytes, which are induced after an autologous transplant of hematopoietic progenitor cells, achieving the remission of the disease. On the other hand, during primary biliary cirrhosis, CD8^+^CD28^−^ T lymphocytes show decreased CD39 expression and fail to inhibit cell proliferation, promoting the severity of this autoimmune disease. In human multiple sclerosis, therapy with IFN-*β* regulates the immune system by reducing autoreactive T cell clones and increases CD8^+^CD25^+^CD28^−^ regulatory T cells. SLE: systemic lupus erythematosus.

As CD4^+^ Tregs, CD8^+^ Treg lymphocyte show reduced levels and function in autoimmune disease patients. In mouse experimental autoimmune encephalomyelitis (EAE) studies, it has been observed that CD8^+^CD28^−^ Treg lymphocytes reduce levels of IFN-*γ* produced by myelin oligodendrocyte glycoprotein-specific CD4^+^ T lymphocytes. In consequence, the expression of costimulatory molecules in antigen-presenting cells interacting with CD4^+^ T lymphocytes is reduced [101]. In this autoimmunity model, there is also a CD8^+^CD122^+^ regulatory T lymphocyte population. This cell population inhibits IL-17, typical of inflammatory process during EAE, and proliferation of CD4^+^ T lymphocytes [102] (Figure 3).

Multiple sclerosis in humans, comparable to EAE in mice, is a disease in which lymphocytes exhibit immune deregulation that is shown as chronic persistent inflammatory response [103]. In that sense, IFN-*β* treatment modulates the immune system, reducing autoreactive T cell clones and increasing CD8^+^CD25^+^CD28^−^ Treg lymphocytes together with plasmacytoid dendritic cells. Treatment with IFN-*β* is highly promising: its use could reduce the activity of the disease [104].

The autologous transplant of hematopoietic progenitor cells in refractory disease systemic lupus erythematosus (SLE) has proven to be highly effective, achieving the remission of the disease. This fact is directly related to the restoration of the CD8^+^FoxP3^+^ Treg lymphocyte population characterized by CD103, PD-1, PD-L1, and CTLA-4 expression. In this case, the function of CD8^+^ Treg lymphocytes on target cells depends on cell-cell contact and TGF-*β* production by regulatory lymphocytes [70]. In addition, CD8^+^CD25^+^FoxP3^+^ regulatory T lymphocytes have been found to be able of suppressing autoantibody production [105] (Figure 3).

Primary biliary cirrhosis is another autoimmune disease that affects humans. In this disease, CD8^+^ Treg lymphocytes express low CD39 and high CD127, a condition that does not change even after culturing the lymphocytes with IL-10. Additionally, the lymphocytes show a deficient suppressant function [106] (Figure 3).

### 1.7. CD8^+^ Treg Lymphocytes in Cancer

**Figure 4:**
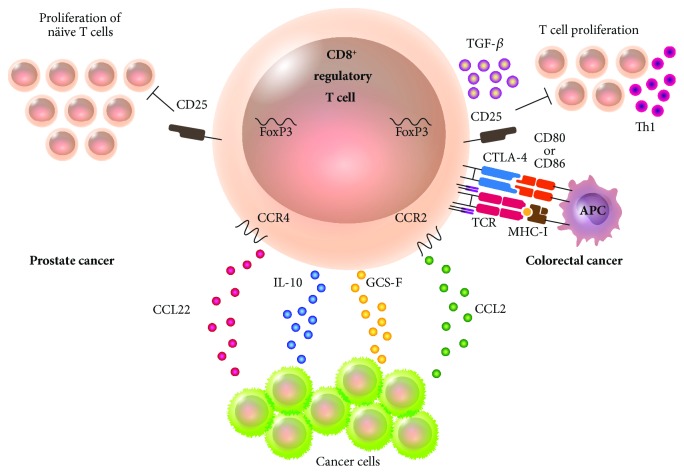
Participation of CD8^+^ Treg lymphocytes in cancer. Regulatory T lymphocytes often aid in the evasion of the immune system by the cancer cell. Specifically, CD8^+^ Tregs can be induced in tumor tissue due to the presence of cytokines as GCS-F and IL-10. They can also be attracted to the tumor after the latter releases the chemokines CCL2 and CCL22 that attract regulatory lymphocytes expressing CCR2 and CCR4. In colorectal cancer, CD8^+^CD25^+^FoxP3^+^ Treg lymphocytes with an immunosuppressive phenotype characterized by expressing CTLA-4 and TGF-*β* inhibit Th1 lymphocyte proliferation. In prostate cancer, CD8^+^CD25^+^FoxP3^+^ Treg lymphocytes have been found to share markers with colorectal cancer and can inhibit naïve T lymphocyte proliferation.

Immune response has been well documented to be altered in cancer. It has been established that antitumoral immune response is avoided by different types of cancer, including kidney, bladder, and colorectal cancer. Antitumoral evasion has been associated to CD8^+^CD28^−^CD127^lo^CD39^+^ lymphocytes [107] (Figure 4). Such lymphocytes can be produced in tumor tissue thanks to the cytokines produced by tumor cells as GCS-F and IL-10. Furthermore, regulatory lymphocytes can be attracted to the tumor because it releases chemokines as CCL2 and CCL22, highly attractive to regulatory lymphocytes expressing specific CCR2 and CCR4. Also, CD8^+^CD28^−^ Treg lymphocytes directly correlate with tumor diagnosis: the higher the concentration of lymphocytes, the worse the diagnosis and vice versa [108]. CD8^+^CD28^−^ T lymphocytes are found at higher levels in advanced stages of non-small-cell lung cancer, maintaining the increase up to the resection of the tumor when there is a decrease in the concentration and the prognosis for the patient is favorable. However, these lymphocytes have yet to be functionally evaluated to confirm whether they were regulatory [109]. In colorectal cancer patients, studies have successfully isolated CD8^+^CD25^+^FoxP3^+^ Treg lymphocytes directly from a tumor. The immunosuppressant phenotype of those lymphocytes is characterized by CTLA-4 expression and TGF-*β* production. They inhibit CD4^+^CD25^−^ T lymphocyte proliferation ex vivo and suppress Th1 cytokine production in themselves [110]. Therefore, these Treg lymphocytes contribute to immune response evasion against tumor and progression of the disease in consequence. In prostate cancer patients, studies have found tumor-infiltrating regulatory lymphocytes with the same phenotype (CD8^+^CD25^+^FoxP3^+^) as the one observed in lymphocytes of colorectal cancer patients. These cells are able to inhibit naïve T lymphocyte proliferation. However, the regulatory activity of these lymphocytes can be reverted by exposing them to TLR-8 ligands as poly-G2. Therefore, the possibility that the manipulation of the TLR-8 signaling pathway can revert immunosuppression mediated by Treg lymphocytes and use it as a therapeutic strategy against cancer is promising [111] (Figure 4). In mice, CD8^+^ Treg lymphocytes have been found as well in cancer induced by inoculation with tumor cell lines. Furthermore, the population CD8^+^CD39^+^Tim-3^+^PD-1^+^LAG-3^+^ has been found to be tumor-infiltrating, produces low levels of IL-2 and TNF, and has a high cytotoxic potential evaluated by granzyme B activity and CD107a mobilization. The expression of CD39 in Treg lymphocytes is created by a recognition of the TCR pathway and promoted by IL-6 and IL-27, which are present in the microenvironment surrounding the tumor. The manipulation of the microenvironment, as well as some therapeutic strategy whose target molecule is CD39, might reduce the evasion of the immune system promoted by Treg lymphocytes and improve the immune response against cancer [112].

### 1.8. CD8^+^ Treg Lymphocytes in Graft-versus-Host Disease

**Figure 5:**
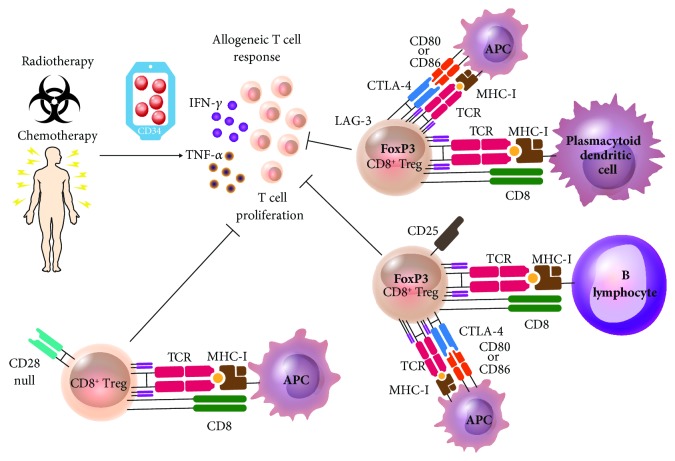
Participation of CD8^+^ Treg lymphocytes in GVHD. After the remission of an oncohematologic disease, patients are treated with chemotherapy and radiotherapy. Later, they receive a hematopoietic stem cell graft from an HLA-compatible donor, at risk of developing GVHD that is characterized by being proinflammatory and producing IFN-*γ* and TNF-*α*. This response can be stopped by CD8^+^ Treg lymphocytes. When they encounter a plasmacytoid dendritic cell, lymphocytes are activated; they acquire phenotype LAG-3^+^FoxP3^+^CTLA-4^+^ and are able to suppress T lymphocyte allogeneic response via CTLA-4. If they are activated by a B lymphocyte, the CD8^+^ Tregs will express CD25^+^CTLA-4^+^FoxP3^+^, which suppresses cell proliferation and release of proinflammatory cytokines. The CD8^+^CD28^−^, a regulatory T cell subpopulation, play a critical role in in vitro and in posttransplantation allogeneic response. They can be generated by in vitro interaction with allogeneic peripheral blood mononuclear cells. Epitope presented in MHC-I is an alopeptide (allogeneic peptide) in all three Treg phenotypes.

CD8^+^ Treg lymphocytes have been described in solid organ transplant and bone marrow transplant as well, which is currently used as hematopoietic stem cell transplantation. In solid organ transplantation, CD8^+^ Treg lymphocytes reduce the risk of transplant rejection in the host by creating host tolerance towards the received tissue or organ [47, 113]. An inverse situation occurs in hematopoietic stem cell transplantation: CD8^+^ Treg lymphocytes participate in the tolerance of donor cells towards the host's tissues. In addition to undergoing ablation of their bone marrow, the host is immunosuppressed by the pharmacological treatment received prior to the transplant and is therefore susceptible to attacks by the immune system cells of the donor. In this situation, the available Treg lymphocytes reduce the risk of GVHD, decreasing the intensity of the damage caused by the donor's cells (Figure 5). As a beneficial collateral effect on the host, a graft-versus-tumor can occur mediated by donor cell, lowering the risk of primary disease relapse. The immunosuppressant effect of Treg cells that prevent GVHD apparently does not compromise the effect of graft-versus-tumor [114, 115]. Still, CD8^+^ Treg lymphocytes are not always found in sufficient quantities, which seems to predispose the patient to GVHD.

Because the inherent immune response to the disease is proinflammatory, the pharmacotherapy given to patients against the illness includes strong immunosuppressants that jeopardize the patient's health since they can lead to infections and/or primary disease relapse. Although the immune response of effector T lymphocytes in the graft versus leukemic cells of the host is needed to prevent relapse, an exacerbated immune response, along with a reduced number of Treg lymphocytes, might cause the death of the host by triggering severe GVHD [116].

This disease causes severe damage in a number of organs, including tissues such as skin, liver, and gastrointestinal tract. It is triggered when immunocompetent donor cells recognize the host cells as foreign and its onset depends on three factors: (1) infused donor cells must be immunocompetent; (2) the host must have antigens absent in the graft; and (3) the host must be unable to generate a response against the graft [117].

Then, why is GVHD generated? It is well known that the main reason of graft rejection in solid organ transplant patients (as in kidney transplant) is high incompatibility between donor and host in HLA histocompatibility. Despite HLA compatibility between donor and host for HLA cells expressing high polymorphism is sought in hematopoietic stem cell transplantation, there may be differences in the HLA showing lower polymorphism that they are not studied routinely. Therefore, foreign antigen recognition after transplant by donor cells is latent and can trigger GVHD [118]. In addition to these risk factors, we must also consider non-HLA genes. An example is that some polymorphisms have been identified in regulatory sequences of genes associated to NK cell KIR receptors. Ligands of KIR receptors are class I HLA molecules. In consequence, the absence of the correct ligands for KIR receptors during hematopoietic stem cell transplanting can lead to cytotoxic activity of the donor NK cells. This can be beneficial to the patient because primary disease relapse is avoided; however, the severity of GVHD is increased as well [119]. Simultaneously, other factors have been related to the development of the disease. Some of them are the source of hematopoietic stem cells (the risk of GVHD is higher when peripheral blood mobilized with growth factors to induce the exit of stem cells is transfused than when bone marrow is transfused), the patient's age (higher risk is associated to older ages), and conditioning of the host with chemotherapy and/or radiotherapy and prophylaxis [117]. These risk factors place GVHD as one of the main causes of failure in hematopoietic stem cell allogeneic transplantation. Nearly 60% of the transplant patients at the Centro Médico Nacional “La Raza” of the Instituto Mexicano del Seguro Social in Mexico City suffer GVHD (unpublished data).

Some hypotheses consider CD8^+^ Treg lymphocytes as responsible for tolerance in the first days after hematopoietic stem cell transplant. This is because, after the transplant, the first T lymphocytes to be present in the peripheral blood are CD8^+^, followed by CD4^+^ lymphocytes in a later stage [120]. Furthermore, recent studies show that when higher concentrations of CD8^+^ T lymphocytes are found in the graft, the possibility of primary disease relapse is reduced without increasing the risk of GVHD. Still, these lymphocytes were not characterized beyond the expression of molecule CD8 on their surface [121].

In GVHD, CD8^+^ Treg lymphocytes have been identified as antigen-specific that are activated when they encounter foreign antigens; that is, they are alloreactive. Their activation is triggered by the encounter of an antigen-presenting cell, like a dendritic cell or a B lymphocyte. In humans, lymphocytes are activated when they encounter a plasmacytoid dendritic cell and acquire a LAG-3^+^FoxP3^+^CTLA-4^+^ phenotype. These cells are able to suppress the allogeneic response of T lymphocytes via CTLA-4 [39]. If the activating cell is a B lymphocyte, the phenotype acquired by the CD8^+^ Treg lymphocyte will be CD25^+^CTLA-4^+^FoxP3^+^. This phenotype suppresses cell proliferation and release of proinflammatory cytokines as IL-1*β*, IL-2, IL-17a, IFN-*γ*, and TNF-*α* by autologous peripheral blood mononuclear cells; CTLA-4 is the molecule with the most involvement in this suppressant function [115] (Figure 5). During the follow-up after a year, a different population of CD8^+^CD28^−^ Treg lymphocytes was observed to be increased and constant *in vivo* in patients that were infused with allogeneic donor cells, using B7-blocking reagents like CTLA-4-Ig that inhibit CD28-B7 together with CTLA-4-B7 interactions as immunosuppressive agent. All the patients survived without showing GVHD [10]. After an allogeneic hematopoietic stem cell transplant, CD8^+^CD28‑ T lymphocytes are found in increased percentage in the patient (Figure 5). These lymphocytes are antigen-specific for tumors related to leukemia patients in remission. Additionally, their proliferation and degranulation are stopped and they become senescent with short telomeres [122].

In human *in vitro* experiments in which the allogeneic condition occurring in a transplant was simulated, CD8^+^CD25^−^ T lymphocytes of a donor were incubated together with dendritic cells of a different donor. This culture yielded CD8^+^CD25^+^FoxP3^+^ Treg lymphocytes that were able to inhibit the allogeneic immune response without affecting the one against the cytomegalovirus, a risk of infection among patients transplanted with hematopoietic stem cells [123]. Another study found that the CD8^+^ cells found in higher concentrations in patients without GVHD expressed FoxP3^+^, unlike GVHD patients. The latter exhibited higher levels of IFN-*γ*-producing Tc1 and IL-17-producing Tc17 lymphocytes [124].

In mice, CD8^+^FoxP3^+^ lymphocytes are the most relevant population and are sufficient to decrease the severity of GVHD [125, 126]. These mouse lymphocytes express the transcription factor FoxP3 and GITR, CD62L, CD28, and CTLA-4 molecules. They produce lower levels of IL-10 and IL-17 and higher concentrations of IFN-*γ*. Additionally, they inhibit CD4^+^ and CD8^+^ T lymphocyte proliferation and expression of costimulatory CD40, CD80, and CD86 molecules during antigenic presentation by dendritic cells [126].

Although the direction of the immune response during graft rejection is inverse to the one present during GVHD, it is also caused by an exacerbated immune response. According to evidence, this response can be controlled by CD8^+^ Treg lymphocytes. In that regard, different subpopulations of CD8^+^ Treg lymphocytes have been described in solid organ transplantation. For instance, the human kidney is not rejected when the percentage of CD8^+^CD28^−^ and CD4^+^CD25^+^FoxP3^+^ Treg lymphocytes increases during the first six months after the transplant [127].

On the other hand, CD8^+^CD122^+^PD-1^+^ Treg lymphocytes reduced rejection to skin graft in mice. These lymphocytes exert a regulatory activity independently from FasL-Fas and thus promote effector CD3^+^ T lymphocyte apoptosis. The inhibition of effector T lymphocyte proliferation depended on IL-10 [47].

A CD8^+^ Treg lymphocyte subpopulation recently described in rats is specific for at least two allogeneic class II MHC peptides in a heart transplant model [128]. This subpopulation shows a low expression or absence of CD45RC (CD45RC^lo/−^) [63, 128]. These lymphocytes exert a regulatory action through IL-34 that they produce. This cytokine acts generating regulatory macrophages from monocytes, promoting in turn the suppressor activity of CD8^+^CD45RC^low^ T lymphocytes. Apparently, CD8^+^CD45RC^lo/−^ lymphocytes have a regulatory activity only when they are the result of blocking the interaction CD40–CD40L (CD8^+^CD40lg) since they produce more IL-34 than naïve CD8^+^CD45RC^lo/−^ lymphocytes (spleen), which are positive to FoxP3. Their regulatory activity can be proven by their ability to inhibit effector CD4^+^CD25^−^ T lymphocyte proliferation, which was induced by IL-34 in a dose-dependent manner. When *in vivo*, these lymphocytes extended the acceptance of the allograft while the production of antibodies against the graft was inhibited [63]. This might constitute a therapeutic strategy to reduce the fatality of acute GVHD in humans, as proven by the use of human anti-CD45RC antibodies in humanized mice [129].

Those CD8^+^ Treg lymphocyte populations that mediate solid organ transplant rejection in GVHD are likely to play a key role in decreasing acuteness of GVHD and promoting the graft-versus-tumor effect.

### 1.9. Concluding Remarks

Although CD8^+^ lymphocytes are described to have an immunosuppressive action, CD4^+^ lymphocytes have been more thoroughly characterized, becoming the model to describe CD8^+^ Treg lymphocytes. No exclusive markers have been described for any of these regulatory lymphocyte populations. For this reason, more than one criterion has been employed to characterize and identify them. The three requisites that must be met to identify CD8^+^ Treg lymphocytes are as follows: (1) they must express more than one marker indicating regulation. (2) They must produce anti-inflammatory cytokines as IL-10 and/or TGF-*β*, and (3) they must inhibit the proliferation of CD4^+^ and/or CD8^+^ effector T lymphocytes. Although FoxP3 is a less abundant marker for CD8^+^ Treg lymphocytes when compared against CD4^+^ Treg, it is relevant to CD8^+^ Treg identification.

The markers that have been described are useful to group Treg lymphocytes in different subpopulations according to their characteristics, location, or role in a pathology. In order to be certain of a subpopulation taking part in GVHD regulation, studies should choose the population with the highest number of markers. This would improve the specificity, but populations showing all the markers would be very small. Working with a reduced and insufficient quantity of CD8^+^ Treg lymphocytes would be inconvenient. If the aim is to find an abundant and regulatory population, it would probably be best to look for subpopulation CD8^+^CD28^−^ and check its regulatory activity, seeking anti-inflammatory cytokine production and proliferation inhibition. A thorough characterization is important given that a CD8^+^CD28^−^ T lymphocyte population might also contain effector lymphocytes [130, 131]. In general, if we were to look for CD8^+^ Treg lymphocytes specific of a pathology, we would resort to the information provided, as shown in Table 1.

The benefits of CD8^+^ Treg lymphocyte participation vary between the pathologies in which the cells play a role. In infectious diseases, it is desirable for the lymphocytes to counter the exacerbated inflammation produces as a response to the microorganism to prevent damage in own tissue. However, an increased participation of regulatory cells might contribute to the pathogen's evasion of the immune response generated by the host and the consequent persistence of the parasite. As shown in Figure 2, CD8^+^ Treg lymphocytes that express ectoenzyme CD39 produce adenosine, which suppresses immune response against two agents: one viral and one bacterial. On the other hand, CD8^+^ Treg lymphocytes allow for the establishment of a parasitic and a viral agent, through IL-10.

Although they are present in some autoimmune diseases and show a presumptive regulatory phenotype, lymphocytes express their regulatory molecules at low levels. In consequence, the molecules are not effective to inhibit lymphocytes and innate immune response cells, responsible for triggered autoimmune inflammatory response. However, effector CD8^+^ Treg lymphocytes inhibiting autoantibody production have been identified in systemic lupus erythematosus (Figure 3).

In cancer progression, CD8^+^ Treg lymphocytes exhibit higher levels and seem to be a tumor-mediated immunosuppressive strategy. They are attracted to the tumor and their permanence is promoted thanks to the evasion of the immune response that might eradicate cancer cells (Figure 4).

Finally, two events occur after an allogeneic hematopoietic stem cell transplant. The first one is GVHD, which can be exhibited in four stages, according to its severity (being 4 the most severe stage). On the other hand, there is the desired graft-versus-tumor effect, in which a strong participation of CD8^+^ Treg lymphocytes is not convenient since it would allow for the reestablishment of the primary disease.

Some *in vitro* studies have obtained CD8^+^CD28^−^ Treg lymphocytes by stimulation of the microenvironment of the cells after an allogeneic transplant, inducing alloanergized CD8^+^ Treg cells. Furthermore, these same markers have been found in increased lymphocyte populations of transplant patients induced to tolerance with belatacept, an immunosuppressant from a fusion molecule bound to CTLA-4. These data define this as one of the ideal cell populations to be studied in allogeneic hematopoietic stem cell transplantation [10]. However, this is not the only CD8^+^ Treg lymphocyte subpopulation involved in the modulation of the immune response in GVHD. Those CD8^+^ Treg lymphocytes with CTLA-4-mediated suppressor activity that are induced by B lymphocytes and plasmacytoid dendritic cells are effective against an allogeneic response (Figure 5).

The study of CD8^+^ Treg cells is not yet complete. A detailed analysis of their identification, regulation mechanisms, and ways of induction, among other events, will allow researchers to know the proportion of CD8^+^ Treg and CD4^+^ effector lymphocytes. This will allow for a cell therapy to prevent and cure infectious and autoimmune diseases as well as cancer and GVHD.

## Figures and Tables

**Table 1:** Phenotypes of CD8^+^ Treg lymphocyte populations and their role in different pathologies.

Pathology	Agent/condition	Phenotype of CD8^+^ regulatory T lymphocytes	Exert suppressive action	Model	References
Infection	Mycobacteria	CD25^+^FoxP3^+^CD39^+^	Inhibit Th1 lymphocyte proliferation	Human	[93, 94]
	HIV	CD28^−^CD127^lo^CD39^+^	Inhibit mononuclear cell proliferation	Human	[97]
	Epstein-Barr virus	FoxP3^+^	Inhibit CD4^+^ T lymphocyte proliferation and produce IL-10	Human	[98]
Autoimmune disease	EAE	CD28^−^	Reduce amount of IFN-*γ* produced by CD4^+^ T lymphocytes	Mouse	[101]
	EAE	CD122^+^	Inhibit characteristic IL-7 production of inflammatory process during EAE; inhibit CD4^+^ T lymphocyte proliferation	Mouse	[102]
	Multiple sclerosis	CD8^+^CD28^−^CD39^+^CD127^−^	Inhibit proliferation		[103, 104]
	SLE	FoxP3^+^	Regulate by TGF-*β*	Human	[70]
	SLE	CD25^+^FoxP3^+^	Suppress production of autoantibodies	Human	[105]
	Primary biliary cirrhosis	CD28^−^CD39^+^CD127^−^	Suppress proliferation	Human	[106]
Cancer	Colorectal cancer	CD25^+^FoxP3^+^	Inhibit CD4^+^CD25^−^ T lymphocyte and Th1 cytokine production	Human	[110]
	Prostate cancer	CD25^+^FoxP3^+^	Inhibit naïve T lymphocyte proliferation	Human	[111]
	Inoculation with tumor cell lines	CD39^+^Tim-3^+^PD-1^+^LAG-3^+^	Exert cytotoxic activity	Mouse	[112]
GVHD	Allogeneic cells	LAG-3^+^FoxP3^+^CTLA-4^+^	Suppress allogeneic response via CTLA-4	Human	[39]
	Allogeneic cells	CD25^+^CTLA-4^+^FoxP3^+^	Inhibit cell proliferation and release of cytokines as IL-1*α*, IL-17a, IFN-*γ*, and TNF-*α*	Human	[115]
	Allogeneic cells	CD28^−^	Inhibit CD4^+^ T lymphocyte proliferation	Human	[10]
	Allogeneic cells	CD25^+^FoxP3^+^	Inhibit allogeneic response	Human	[123]
	Allogeneic cells	FoxP3^+^	Inhibit CD4^+^ and CD8^+^ T lymphocyte proliferation and CD40, CD80, and CD86 expression in CD	Mouse	[125, 126]

GVHD: graft-versus-host disease; HIV: human immunodeficiency virus; EAE: experimental autoimmune encephalomyelitis.
